# Antibody performance in ChIP-sequencing assays: From quality scores of public data sets to quantitative certification

**DOI:** 10.12688/f1000research.7637.2

**Published:** 2016-03-11

**Authors:** Marco-Antonio Mendoza-Parra, Vincent Saravaki, Pierre-Etienne Cholley, Matthias Blum, Benjamin Billoré, Hinrich Gronemeyer

**Affiliations:** 1 Institut de Génétique et de Biologie Moléculaire et Cellulaire (IGBMC), Equipe Labellisée Ligue Contre le Cancer, Department of Functional Genomics and Cancer, Centre National de la Recherche Scientifique UMR 7104, Institut National de la Santé et de la Recherche Médicale U964, University of Strasbourg, Strasbourg, 67404, France

**Keywords:** ChIP-sequencing, antibody, quality, massive parallel sequencing

## Abstract

We have established a certification system for antibodies to be used in chromatin immunoprecipitation assays coupled to massive parallel sequencing (ChIP-seq). This certification comprises a standardized ChIP procedure and the attribution of a numerical quality control indicator (QCi) to biological replicate experiments. The QCi computation is based on a universally applicable quality assessment that quantitates the global deviation of randomly sampled subsets of ChIP-seq dataset with the original genome-aligned sequence reads. Comparison with a QCi database for >28,000 ChIP-seq assays were used to attribute quality grades (ranging from ‘AAA’ to ‘DDD’) to a given dataset. In the present report we used the numerical QC system to assess the factors influencing the quality of ChIP-seq assays, including the nature of the target, the sequencing depth and the commercial source of the antibody.  We have used this approach specifically to certify mono and polyclonal antibodies obtained from Active Motif directed against the histone modification marks H3K4me3, H3K27ac and H3K9ac for ChIP-seq. The antibodies received the grades AAA to BBC ( www.ngs-qc.org). We propose to attribute such quantitative grading of all antibodies attributed with the label “ChIP-seq grade”.

## Introduction

Chromatin immunoprecipitation combined with massive parallel sequencing (herein described as ChIP-sequencing or ChIP-seq) is currently intensively used as a method for assessing protein-DNA interactions and/or chromatin modifications on a genome-wide scale. It is well-accepted that optimal ChIP-sequencing assays require highly specific and sensitive antibodies, thus an important battery of validation tests is strongly recommended for their characterisation ^1^. In addition to the concerns of the scientific community about the current strategies for validating antibodies, which are commercially promoted for certain various applications, also the assessment of antibody performances in ChIP-sequencing assays remains still a major issue, mainly due to the absence of quantitative approaches for qualifying ChIP-seq assays.

We have previously described a universal *in silico* approach to generate quality descriptors for any ChIP-sequencing and related datasets ^2^. Importantly, this concept has been used to establish the largest database worldwide, which harbors currently the quality scores for more than 28,000 publicly available datasets ( www.ngs-qc.org). Notably, in contrast to other metrics previously described for the qualification of ChIP-seq assays ^3^, this system evaluates the robustness of the enrichment patterns populating a given profile by comparative analyses of the original profile and profiles generated after random sub-sampling from a reduced fraction of the total mapped reads. This methodology is applicable to any type of enrichment-related dataset and the inferred quality indicators can thus be used for comparative purposes. In order to provide simple and intuitive quality designations the quality indicators were discretised using a three letter grading score; accordingly, the profiles range from highest “AAA” to the lowest “DDD” quality.

In this study, we present first an analysis concerning the quality of the available >28,000 data sets in the context of their sequencing-depths used and of the antibody sources. Thereafter, we introduce a certification procedure, which should be used to associate a defined referenced batch of an antibody with a reliable validated ChIP-sequencing grade.

## Materials and methods

### NGS-QC database content

All datasets presented in the retrospective analysis were originally retrieved from the GEO database and processed with the NGS-QC Generator algorithm. Quality indicators (QCis) were computed as previously reported ^2^. Briefly, QCis were generated by comparing the original read intensity profile with those observed in a fraction of the total mapped reads. For this, total mapped reads (TMRs) were first randomly sub-sampled at three defined subsets (90%, 70% and 50% respectively), then the read counts in 500 nt bins of the genome were computed for each of the random sub-sampled as well as in the original dataset. In the ideal theoretical case the read counts in all genomic bins are expected to decrease proportionally to the random subsampling (e.g., a 50% decrease of the read count intensity (RCI) when 50% of the original TMRs were sub-sampled). Genomic regions presenting the lowest variations from this theoretically expected value are considered “robust” to the random sampling and thus of high quality. For quantitation we calculated the fraction of genomic windows with RCI dispersions within defined levels (2.5, 5 and 10%).

Quality scores for all analysed publicly available datasets are available at www.ngs-qc.org. The antibody references associated with the ChIP-seq datasets analysed in this study are given in Table 1.

**Table 1.** Antibody source information for ChIP-seq datasets presented in this study as recovered from GEO.

Target Molecule	Antibody vendor	Number of references	Antibody ID
H3K27me3	Abcam	3	ab6002, ab4729, ab39155
H3K27me3	Active Motif	6	AM39155, AM39536, AM61017, AM39535, AM39133, AM39156
H3K27me3	Bethyl Laboratories	1	A302-175A
H3K27me3	Cell Signaling	3	9733S, 9733, 9773S
H3K27me3	Diagenode	1	pAb-069-050
H3K27me3	Millipore	6	07-449, 17-622, 07-499, ABE44, 07-450, 07-473
H3K27me3	Santa Cruz Biotechnology	1	sc-2003
H3K27me3	Wako	1	301-95253
H3K27ac	Abcam	3	ab4729, Ab4729, ab4739
H3K27ac	Active Motif	4	AM39133, AM39135, AM39297, AM39134
H3K27ac	Millipore	1	07-449
H3K27ac	Upstate	1	07-360
H3K27ac	Wako	1	306-34849
H3K4me3	Abcam	3	ab8580, ab1012, ab8895
H3K4me3	Active Motif	3	AM39159, AM35159, AM39160
H3K4me3	Cell Signaling	4	9751S, 9751, 9751B, 9727S
H3K4me3	Diagenode	1	pAb-003-050
H3K4me3	Millipore	8	07-473, 04-745, 17-614, 05-745, 07-449, 17-678, 07-473CA, 07-474
H3K4me3	Santa Cruz Biotechnology	1	sc-48790
H3K4me3	Wako	3	307-34813, 301-95253, 302-95283
H3K4me1	Abcam	4	ab8895, ab8899, ab1012, ab1791
H3K4me1	Active Motif	3	AM39297, AM39298, AM39635
H3K4me1	Cell Signaling	1	9723S
H3K4me1	Diagenode	2	pAb-037-050, pAb-194-050
H3K4me1	Millipore	1	07-436
H3K4me1	Santa Cruz Biotechnology	1	sc-265

### Antibody certification procedure

We propose the following certification procedure for antibodies to be used in ChIP-seq and related enrichment-based technologies. Clearly, this certification will not replace but rather complement the molecular biology assays currently used for validating antibody specificity. Indeed, it provides a certificate of antibody performance specifically in ChIP-seq assays. The following experimental conditions were used for the certification of antibodies described in this study:

***Cell culture.*** HeLa cells were grown in DMEM 1g/L glucose, 5% Fetal Calf Serum and 40µg Gentamicin to a density of 15–20 millions cells/15cm plates. Cells were fixed for 30min with paraformaldehyde (1% in PBS). Fixation was quenched with 0.2M glycine in PBS, then cells were washed three times with PBS, collected and stored at -80°C.

***Chromatin immunoprecipitation.*** **Sonication:** 40 million cells were sonicated in 500µL of Lysis Buffer (1% Na-deoxychlorate, 50mM TrisHCl pH8, 140mM NaCl, 1mM EDTA, 1% Triton X-100) containing 5-times diluted Protease Inhibitor Cocktail (PIC; Roche Diagnostic; 1 tablet solubilized in 10ml Lysis Buffer). Sonication was performed with a Bioblock Scientific instrument (Vibra Cell 75043; 40 cycles, 30s ON end 59s OFF; 38% power). Chromatin fragmentation was evaluated by agarose gel electrophoresis as follows: 20µL of sonicated chromatin was diluted with 20µL TE (10mM Tris-HCl pH 8, 1mM EDTA) and 5µL 5M NaCl was added. Diluted chromatin was incubated at 100°C for 30 min, centrifuged at 12,000 rpm at room temperature and the supernatant was loaded onto a 2% agarose gel.

**Chromatin Immunoprecipitation:** 25µL of ChIP-IT Protein G Magnetic Beads (ActiveMotif) were incubated with the antibody under evaluation (the amounts of antibody in use corresponded to that indicated by the supplier’s information) in presence of 100µL PIC-containing Lysis Buffer. After two hours at 4°C on a rotating shaker, chromatin from 3 million cells was added and the final volume was adjusted to 500µL with PIC-containing Lysis Buffer and incubation on a rotating shaker was continued overnight at 4°C. The immunoprecipitated chromatin was recovered by magnetic bead separation, followed by multiple washing steps on a custom liquid handling platform (TECAN EVO75). Specifically, the washing is performed as follows: (1) Low salt washing (0.1% SDS, 1% Triton X-100, 2mM EDTA, 20mM TrisHCl pH 8, 150mM NaCl); (2) High salt washing (0.1% SDS, 1% Triton X-100, 2mM EDTA, 20mM TrisHCl pH 8, 500mM NaCl); (3) LiCl-washing (0.25M LiCl, 1% IGEPAL CA630, 1% Na-deoxycholate, 1mM EDTA, 10mM Tris pH 8) and (4) 1×TE washing. The immunoprecipitated chromatin was eluted and de-crosslinked in 100µL of elution buffer (1% SDS, 100mM NaHCO _3_, 250mM NaCl, 0.2mg/ml Proteinase K) and incubated for 4 hours at 65°C. The eluted chromatin was supplemented with 200µL H _2_O and 300µL phenol/chloroform/isoamyl alcohol (25/24/1) mix was added. After two extraction steps, the aqueous phase was subjected to ethanol precipitation in presence of 1µL GlycoBlue (Invitrogen; 15mg/ml). The precipitated material was re-suspended in 45µL H _2_O; 5µL was used for validation by quantitative PCR, the remaining 40µL was used for DNA library preparation.

***DNA library preparation and massive parallel sequencing.*** The DNA library preparation for massive parallel sequencing was performed according to standard procedures (NEXTFlex ChIP-Seq Kit (Biooscientific)) adapted to automation by our custom liquid handling platform (TECAN EVO75). Prior to DNA sequencing library preparation was monitored using a Tapestation (Agilent). Samples were sequenced on an Illumina HiSeq2500 platform following manufacturer’s standard procedures.

***NGS-QC certification.*** The antibody certification is based on the quality control system previously described ^2^. The certification is based on two biological replicate ChIP-seq assays performed at high sequencing depths (~50 million mapped reads per dataset). For each replicate the global quality grades were computed. In addition the following issues were part of the certification:

**Optimal sequencing depth:** This is performed by re-computing quality grades at decreasing fractions of the initial total mapped reads (TMRs). Briefly, defined TMR subsets were generated by random sampling (20%, 40%, 60%, 80% and 100% of TMRs) and quality scores were assessed. The sequencing depth at which the quality grades transit from A to B is extrapolated and designated as optimal sequencing depth.

**Local QC Irreproducibility Discovery Rate (local QC-IDR):** Concordance among biological ChIP-seq replicate assays were previously assessed by the Irreproducibility Discovery Rate assay ^4^. Similarly, we have established an IDR-type assay based on the comparison of the location of genomic regions (500nt length) presenting the lowest read count intensity dispersion (dRCI). Briefly, genomic regions displaying a dRCI <10% in the two biological replicates were paired and ranked on the basis of their lower absolute difference between paired dRCI values (genomic regions present in only one dataset are kept and paired with a “penalty genomic window” for which a dRCI=15% was allocated). Finally, the local QC-IDR was defined as the fraction of the top 5,000 windows using a sliding window of 500nt.

***Comparing QC scores with those assessed for publicly available data.*** Antibody certification scores were compared with quality scores computed from publicly available data in the context of their TMRs. For it, a scatter-plot displaying quality scores (y-axis) relative to the TMRs (x-axis) was generated for all datasets associated to the particular target molecule, as well as for that related to the certified antibodies.

## Results

### Contents of the NGS-QC database

**Figure 1.**
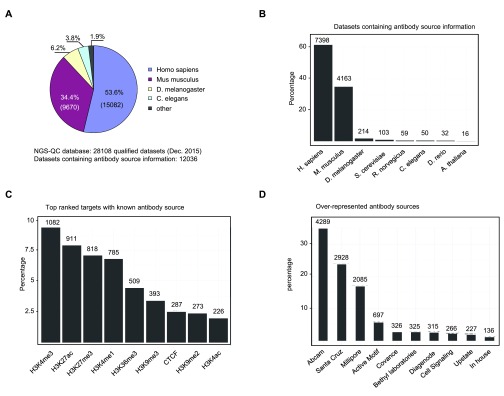
Current content of the NGS-QC Generator database. ( **A**). The NGS-QC Generator database hosts currently quality scores for 28,108 datasets from a variety of organisms. For about 50% of the datasets the commercial source of the used antibodies could be retrieved from the information present in GEO. ( **B**). Classification per organism of the qualified datasets for which the antibody source is known. Note that nearly 90% correspond to Homo sapiens or Mus musculus datasets. ( **C**). Top ranked target molecules for which the antibody source is known. ( **D**). Ten most-used commercial antibodies retrieved from the NGS-QC database.

The NGS-QC Generator database hosts currently quality scores for >28,000 datasets ( www.ngs-qc.org; December 2015), covering a variety of ChIP-sequencing and related assays (e.g. Dnase-seq, FAIRE-seq, MBD-seq, GRO-seq, etc) performed from 9 species (Homo sapiens: 54%; Mus musculus: 34%; D. melanogaster: 6%; etc) ( Figure 1A). As it is based on datasets available from GEO, we retrieved antibody sources for 12,036 datasets from the complementary information; 96% of these corresponded to human or mouse-related assays ( Figure 1B). Furthermore, these datasets concern 680 different target molecules, with several histone modifications being highly represented ( Figure 1C). Finally, thanks to the information provided by the authors, we managed to trace the commercial sources of the antibodies ( Figure 1D). Overall, this analysis reflects the interest of the scientific community in studying the role of epigenetic factors in human and mouse systems. The data reveal also that three companies dominate the market, as they provided the antibodies for 75% of the evaluated datasets.

### ChIP-seq quality relative to sequencing-depth and antibody source

As ChIP-seq datasets for the histone modifications H3K4me3, H3K27ac, H3K27me3 and H3K4me1 are the most represented in the collection ( Figure 1C), we focused our analyses on these datasets.

**Figure 2.**
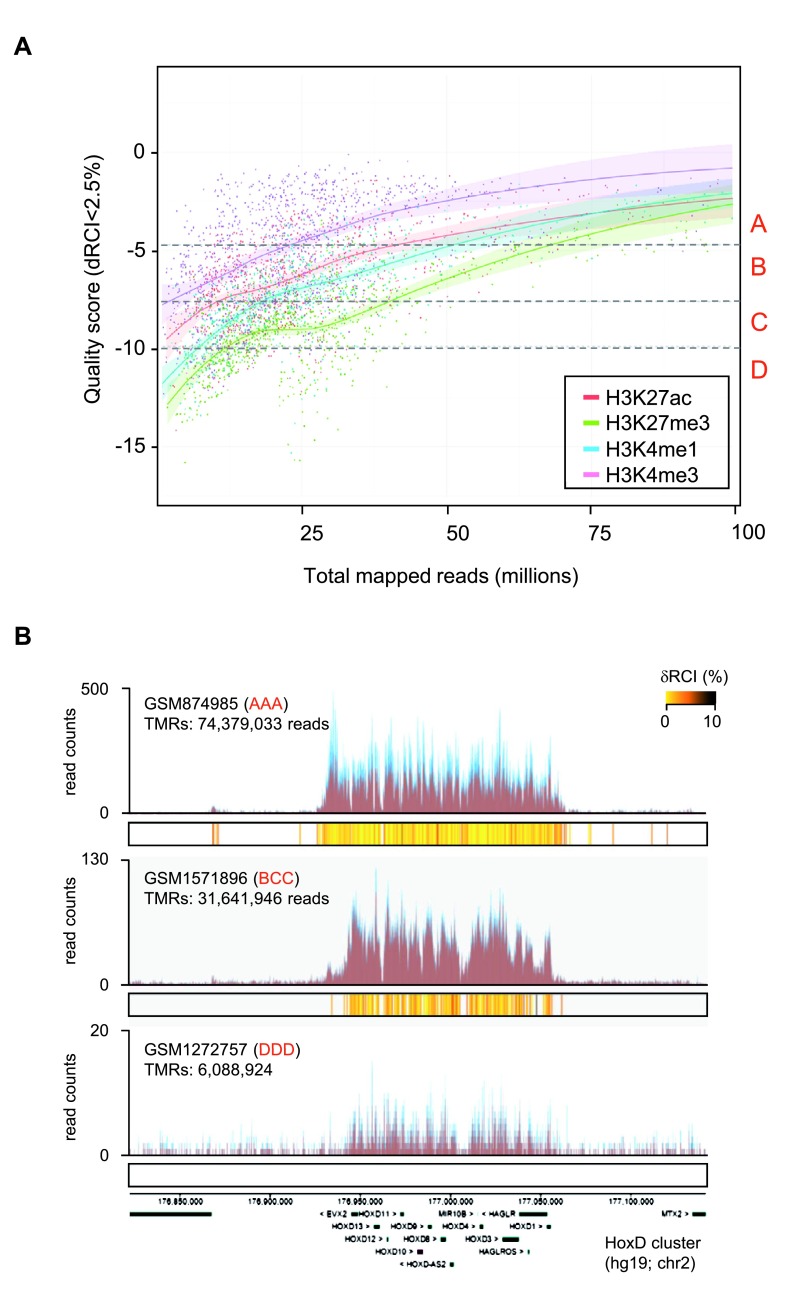
Influence of the sequencing-depth on the quality of ChIP-seq assays. ( **A**) Scatter-plot illustrating global quality scores relative to sequencing depth for ChIP-seq assays concerning the indicated histone modification marks. All illustrated mouse or human datasets were retrieved from the public domain and qualified with the NGS-QC Generator. Quality scores were computed for a read count intensity dispersion (dRCI) of 2.5%. Broken lines depict to the transition borders between discretized quality grades (“A”, “B”, “C” or “D”) defined as the quartiles of the quality distribution computed from the entire NGS-QC collection (>28,000 datasets). The continuous lines correspond to the locally weighted scatter-plot smoothened (LOWESS) regression curves (displayed confidence interval p-value: 0.995). ( **B**) Local genomic view (HoxD cluster) displaying read count intensity patterns for the histone modification mark H3K27me3 derived from three different datasets. In all cases the same cell type (human ES cells) and antibody source (Millipore: # 07–449) has been used, while different DNA sequencing coverage was applied. Note that the associated quality grades correlate with the sequencing depth.

We first evaluated quality grades in the context of sequencing depths. Importantly, the expected increase in quality relative upon increased sequencing-depth is not uniform but is rather target-dependent, as is supported by the different slopes of the datasets ( Figure 2A). Indeed, while H3K4me3 datasets reach high quality even with relatively low sequencing depth (~25M TMR), the profiles for H3K27me3, which display broad signals, require >60M TMRs for reaching an “AAA” qualification ( Figure 2A). Figure 2B illustrates the differences in quality that correspond to the different quality grades ( Figure 2B); note that these data have been obtained with human embryonic stem (ES) cells using the same antibody ^5– 7^.

Another aspect to highlight is the fact that there is a high variability of quality scores for profiles from <50M TMRs. In contrast, all datasets with higher sequencing depths tend to have quality grades between “B” and “A” but the number of such datasets is low compared to those with less sequencing coverage. These observations clearly support the notion that in addition to the sequencing depth, other experimental factors - including the commercial source of the antibody - directly influence the global quality of ChIP-sequencing assays.

**Figure 3.**
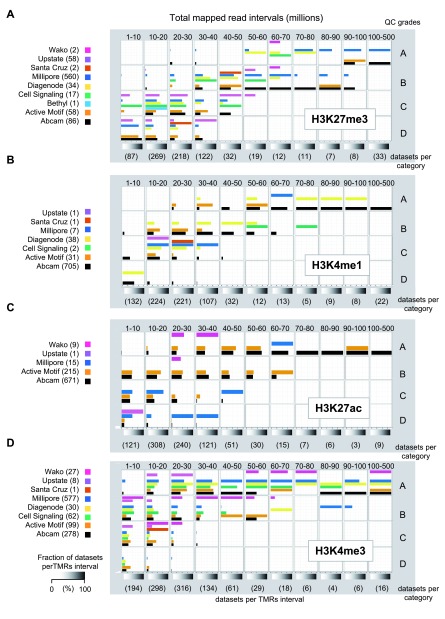
Bar graphs illustrating the frequency of datasets related to a defined antibody vendor per quality grade and total mapped reads (TMRs) interval. Datasets for each target were categorized on the basis of their sequencing coverage (X-axis: from 1 to 100 total mapped million reads; intervals of 10 million and a last category from 100–500 million), as well as on their quality grade (Y-axis: from “A” to “D”, defined on the basis of a read count intensity dispersion (dRCI) threshold criterion of 2.5%). The illustrated bar graph correspond to the fraction of datasets related to a given vendor per TMRs interval. **A** to **D**. Frequency bar graphs corresponding to H3K27me3, H3K4me1, H3K27ac and H3K4me3, respectively.

To further explore the role of the antibody source, we have classified the different datasets for antibody vendors and TMR intervals, such that the quality grades per datasets are displayed in context of these two parameters. As illustrated in Figure 3, this classification recapitulates the influence of the sequence depth irrespective of the antibody source and reveals the target–specific quality differences. For example at <50 million TMRs most of the evaluated H3K27me3 datasets ( Figure 3A) have quality grades lower than “A”. However, while datasets generated with an antibody from a particular vendor would rapidly gain in quality grades with increased TMRs, those from other sources improve only weakly and require much more TMRs to reach the highest quality grades; examples are the H3K27me3 antibodies of Millipore and Abcam with the Millipore one exhibiting the higher robustness. Similarly, H3K4me1 datasets require >20M TMRs to reach high quality grades but a few antibodies yielded datasets at 20–30M TMRs that got grade “A”, while others needed much higher coverage to reach “A” grades; examples are again the Abcam and Millipore antibodies, which show the inverse robustness of their H3K27me3 counterparts ( Figure 3B). Notably, datasets related to the histone modification marks H3K27ac and H3K4me3 present high quality grades even for lower TMRs. In fact a minimum of 10–20 million reads was sufficient to attribute quality grade “A” to some H3K27ac datasets ( Figure 3C) and even lower DNA sequencing coverage was required to reach such quality scores for H3K4me3 datasets ( Figure 3D).

Taken together this analysis reveals that while there is a general direct correlation between the sequencing depth and the quality scores of the corresponding profiles, there are three important considerations: (i) a minimal sequencing depth is required to ensure an acceptable quality of the assay; (ii) the nature of the histone mark has a profound impact on the sequencing depth needed to reach high quality grades, as the increase in quality with increasing coverage is not genuine and differs between marks; and (iii) the source of the antibody has a significant impact on the quality of the profile that can be obtained at a certain sequencing depth.

Overall, this *meta* analysis provides not only numerical values to assess the effect of the sequencing depth on ChIP-seq quality, but it offers in addition the possibility to rate the performance of new antibodies and antibody batches. Note, however, that a multitude of additional factors affects the quality of ChIP-seq datasets independently of the antibody source; these factors include cell types/tissues in use, the chromatin fixation conditions, experimenter variability, and many more. Indeed, the scatter of QCis observed at defined TMR intervals ( Figure 2A) results most likely from such effects. As a consequence antibody certification for ChIP-seq assays should be based on standardized experimental conditions together with quality assessment derived from biological duplicates at high sequencing depth, such that optimal sequencing depths can be recommended to the users.

### Improved certification for “ChIP-seq grade” antibodies

To respond to the needs of the scientific community for highly reliable, target specific, low background polyclonal (and monoclonal) antibody sources for epigenome or any other ChIP-based studies commercial antibody suppliers have incorporated “ChIP-seq grade” antibodies in their portfolio and based this grade on visual inspection of generally a single selected genomic region from a ChIP-seq profile. However, there are several problems associated with this procedure: First, the screenshot of a rather short genomic region says next to nothing about the quality of the entire genome-wide profile. Second, the assay conditions and sequencing statistics are generally not provided, and third, information on replicate and batch variation is generally missing.

**Figure 4.**
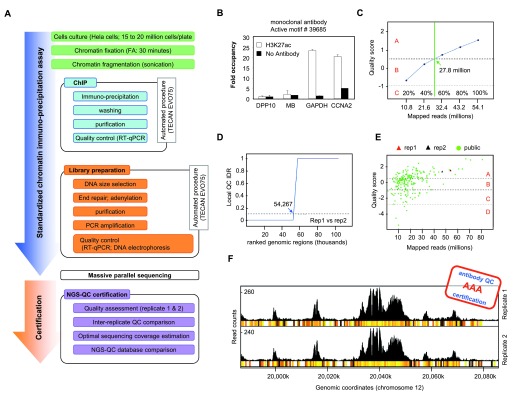
A quantitative approach for the certification of ChIP-seq grade antibodies. ( **A**). Scheme of the antibody certification procedure comprising the standardization of the experimental steps involved in sample preparation and computational treatment of ChIP-seq datasets. ( **B**) to ( **F**). Examples of some of the key analytical data generated from biological replicates during the certification process using the Active Motif anti-H3K27ac antibody # 39685. ( **B**). Quantitative RT-PCR validation of the chromatin enrichment efficacy performed at the end of the standardized experimental procedure. DPP10 and MB (Myoglobin) refer to reference regions devoid of H3K27ac, while GAPDH and CCNA2 promoter regions are used for the evaluation of the enrichment levels (Fold occupancy relative to DPP10). ( **C**). Extrapolation of the optimal sequencing depth by sub-sampling of the initial TMRs. The inferred quality scores are displayed relative to the mapped reads. The vertical green line defines the reads at which the transition of quality grade from “A” to “B” is observed. ( **D**). Irreproducibility discovery rate (IDR) of biological replicates. The local QC IDR is defined as the fraction of genomic regions (500nt window size) which exhibit reproducibly read count intensity dispersion levels (dRCI) below 10%. For computation, all genomic regions were subdivided in groups of 5,000 windows (sliding window with a span of 500nt) followed by ranking according to their RCI dispersion. The X-axis displays the ranked genomic regions for which the local QC IDRs (Y-axis) were computed. The broken horizontal line delimits a local QC IDR threshold of 0.1, which is used to define the number of genomic windows with sub-threshold IDR levels. ( **E**). Scatter-plot displaying the quality scores computed during the antibody certification (triangles correspond to the two biological replicates used in the certification process) relative to those of publicly available H3K27ac ChIP-seq datasets. ( **F**). Genomic region (chromosome 12), illustrating the local enrichment of H3K27ac mark generated by the certified antibody. Below each read count intensity profile, a heatmap illustrates the robustness of genomic bins (500nt) to random sampling as measured by their dRCI levels (yellow to black: 0–10%). The certification grade attributed to this antibody is quality grade “AAA” as indicated.

To improve this situation we have set up a certification procedure which is based on (i) the use of a highly reproducible pipeline for performing ChIP-seq samples preparation and (ii) the use of the NGS-QC Generator tool for assessing quality grades from biological replicates that can this time reflect the performance of the antibody ( Figure 4A). The procedure involves an extensive documentation including experimental details, full sequencing statistics and quality analysis of the profiles obtained from biological replicates together with recommendations for how to use this antibody to obtain optimal quality grades.

This certification procedure, which is based on the use of an automated liquid handling platform for ChIP assays and subsequent preparation of the corresponding sequencing library, has been set up with a panel of six antibodies kindly provided by Brian Egan of Active Motif. Specifically, antibodies targeting the histone modification marks H3K4me3, H3K27ac and H3K9ac were enrolled in the certification process; for each of them monoclonal and polyclonal preparations were assessed. ChIP assays were performed with Hela cells cultured and formaldehyde-fixed (see Materials and methods). The efficacy of the ChIP was verified by quantitative RT-PCR ( Figure 4B). The conditions for the automated preparation of the sequencing library were also standardized.

Massive parallel sequencing was performed at high depth such that coverage was not a limiting factor for quality assessment and that the minimal sequencing depth to obtain “A” quality scores could be predicted ( Figure 4C). An important component is the evaluation of the irreproducibility discovery rate of biological replicates as an integral part of the certification process ( Figure 4C). Finally, we also performed a comparison with the quality grades assessed relative to the publicly available datasets ( Figure 4D) and we provide screenshots of several local genomic regions to illustrate the read count enrichment for the biological replicates and to provide a local quality score (500nt window, heatmap; Figure 4E).

Overall, this certification procedure provides quantitative means for assessing antibody performance in ChIP-sequencing assays, thus their "ChIP-seq grade" would not represent a questionable marketing argument but rather a solid certification label.

## Conclusion

As ChIP-sequencing assays are becoming widely used for studies in epigenetics/chromatin modification and the definition of chromatin interactions with transcription factors and other regulatory factors/machineries, it is of highest importance that scientist have access to antibodies of high quality and reliability such that the antibody performance can be excluded as a source of low quality and variability between datasets. Such quality differences are obvious from QC indicator database that we have established ( www.ngs-qc.org). At the time of the revision of this manuscript, this database comprises more than 30,000 qualified datasets and this number is expected to increase largely within the years with the “democratisation” of this technology. It is important to point out that the present retrospective analysis revealed that an important fraction of the datasets in the public domain is below acceptable quality standards to perform for example multi-profile comparisons.

Here we clearly demonstrate, on the basis of a numerical quality evaluation of a large number of datasets, several features which impact on ChIP-seq quality. First, there is a direct correlation with the sequencing depth. Second, we reveal the direct influence of the nature of the molecular factor as another parameter to consider for the minimal sequencing depth that has to be used to get high quality datasets. As a rule of thumb transcription factors and histone modifications that produce locally confined signals (“sharp peaks”) in the corresponding profiles can be sequenced at lower coverage that for example histone marks which generate “broad peaks”. Third, given that analysis is based on a rather comprehensive collection of datasets, we were for the first time in a position to evaluate the effect of the commercial source of an antibody on the quality of the obtained datasets. Importantly, while we did not observe dramatic differences among the various antibodies at high sequence coverage, there are indeed significant differences when sequencing was performed at lower coverage. Fourth, there is a large difference between the quality of experiments even when the same antibody and the same cell type is used; most plausibly this is due to difference in the performance/materials/methods that have been used in the various laboratories. Finally, taken in consideration that an important number of ChIP-sequencing assays are performed with polyclonal antibodies, potential differences between antibody batches could be in principle evaluated via dataset quality assessment. This being said, it is difficult to perform dataset population studies between antibody batches, as this information is not systematically available in public repositories.

For this reason we have developed an antibody certification procedure dedicated to ChIP-sequencing applications, in which (i) sample preparation is performed under standardized conditions; and (ii) quantitative analytical metrics are applied for assessing the antibody performance. With such a certification at hand the experimenter knows the performance of a given antibody and can follow the guidelines for its use to obtain maximal quality at minimal cost.

This methodology has been set up with multiple antibodies provided by Active Motif; the corresponding quality certification reports are freely available from our website ( www.ngs-qc.org). Considering that this approach provides quantitative data for attributing the "ChIP-seq grade" to a particular antibody batch, the use of such metrics will significantly improve consumer confidence in this label. It is worth mentioning that the above proposal for an antibody certification procedure does not imply to imperatively use the specified reagents for library preparation or other steps of the certification procedure but rather emphasizes the need for standardized procedures, which reduce the impact of experimenter-derived variability by the implementation of automated liquid handling steps. In that manner, ChIP-sequencing dedicated antibodies would be optimally evaluated on their performance such that at the end we could call them "ChIP-seq grade" on the basis of quantitative readouts.

## Data access

Quality reports for all ChIP-sequencing datasets discussed in this study are available via the NGS-QC database ( www.ngs-qc.org). Raw data associated to the discussed antibody certification procedure is available at the NCBI Gene expression Omnibus database under accession number GSE76618.
