# Supplementary material for: Antibody performance in ChIP-sequencing assays: From quality scores of public data sets to quantitative certification
Source: F1000Res. 2016 Mar 11;5:54. Originally published 2016 Jan 12. [Version 2] doi: 10.12688/f1000research.7637.2 (PMC4893979; doi:10.12688/f1000research.7637.2)
Supplement: Peer review history

# Peer review: Referee response for version 2

**DOI:** 10.5256/f1000research.8971.r12983

**Published:** 2016-03-22

**Authors:** Antonio Hurtado

**Affiliations:** 1 Breast Cancer Research group, Centre for Molecular Medicine Norway (NCMM), University of Oslo, Oslo, Norway

After reading the comments from the authors, I am satisfied with the additional comments incorporated by Mendoza-Parra *et al. *Therefore I accept the article in the way is currently submitted.

I have read this submission. I believe that I have an appropriate level of expertise to confirm that it is of an acceptable scientific standard.

# Peer review: Referee response for version 1

**DOI:** 10.5256/f1000research.8224.r12202

**Published:** 2016-03-04

**Authors:** Jason S. Carroll, Adam W. Nelson

**Affiliations:** 1 Cancer Research UK Cambridge Institute, University of Cambridge, Cambridge, UK

ChIP-seq is becoming a standard tool for transcription factor or histone mark mapping, but many assumptions are made about the reproducibility of this method, particularly between factors. There is a general assumption that one set of parameters are appropriate for all ChIP-seq experiments, regardless of the factors being purified. No genuine gold standard exists for ChIP-seq and the current accepted guidelines defined by Encode, may or may not be of sufficient robustness (i.e. Encode recommend two ChIP-seq replicates, which eliminates any possibility for statistical differential analysis and simply wouldn’t be acceptable for almost any other genomic analysis). One big issue in ChIP-seq, is the quality of the antibody and the number of required reads to get robust data. This manuscript provides a very useful and important tool for dealing with this critical issue. The QC indicator described and tested in this manuscript, provides a user-friendly way of gaining insight into the best antibody for a factor and the minimum read depth needed for an experiment to capture most binding sites and to therefore be considered successful. The system described here provides information about the best reagents and the appropriate sequencing depth, using a clear and informative grading system. The findings from this paper show that different sources of antibodies have distinct efficiencies and that different sequencing depth is required for individual factors, an importantly and largely unknown finding. Whilst the method is based on *a priori* knowledge and doesn’t account for non-specific antibodies or isn’t appropriate for novel factors that haven’t been previously explored, the QCi approach is an important and much needed resource, for both novices and experts. This tool will undoubtedly improve the quality of the data that is being produced and analysed by the community and this should be the first place to start for anyone thinking of doing ChIP-seq experiments.

We have read this submission. We believe that we have an appropriate level of expertise to confirm that it is of an acceptable scientific standard.

# Response

**Published:** 2016-03-09

**Authors:** Marco-Antonio Mendoza-Parra

**Affiliations:** IGBMC, France

We would like to thank Jason Caroll and Adam Nelson for investing time in the revision of our manuscript. Specially, we are very pleased to learn that we share a common interest in identifying the parameters that impact on the performance of ChIP-seq and related assays. Indeed, it is our aim to describe in this manuscript the usefulness of the NGS QC system, which we have provided freely to the scientific community to infer optimal sequencing depth levels, in addition to providing a resource that specifies the relative performance of previously used antibodies in ChIP-seq assays on the basis of numerical quality indicators. Finally, we describe an antibody certification procedure, which can be used to assess the ChIP-seq “grade” of any existing or novel antibody batch.

# Peer review: Referee response for version 1

**DOI:** 10.5256/f1000research.8224.r12201

**Published:** 2016-02-12

**Authors:** Antonio Hurtado

**Affiliations:** 1 Breast Cancer Research group, Centre for Molecular Medicine Norway (NCMM), University of Oslo, Oslo, Norway

This paper suggest a quantitative approach for certification of ChIP-seq grade antibodies. This is an interesting aim and the authors point out several factors influencing the quality of ChIP-Seq data sets.

In the paper they have retrieved antibody sources for 12036 datasets from GEO, and these data are illustrated in different figures. Using data from GEO they have also produced a figure illustration how different sequencing depth can be needed for different histone modification analyzed. The authors have also used 3 antibodies from Active Motif to illustrate how they can give scoring to a given antibody. However, if the authors aim to make certification of specific antibodies they should provide a more detailed and standardized protocol. Moreover, they should also comment why they choose to x-link 30 min in paraformaldehyde, rather than more common 10 min in 1% formaldehyde. The choice of kit for making library should be discussed, and also why they have used a not so common kit in their procedure.

Also variation in performance between different lot of polyclonal antibodies should be discussed. It would also be interesting with a discussion of the practical relevance of the different grades from scoring for a specific antibody.

I have read this submission. I believe that I have an appropriate level of expertise to confirm that it is of an acceptable scientific standard, however I have significant reservations, as outlined above.

# Response

**Published:** 2016-03-09

**Authors:** Marco-Antonio Mendoza-Parra

**Affiliations:** IGBMC, France

We are very grateful to Antonio Hurtado for reviewing our manuscript, and for his useful comments/suggestions to improve our manuscript. Following the reviewer’s suggestions, we have included additional elements in the revised version of the manuscript. Specifically we would like to emphasize that the described certification procedure does not imply to imperatively use a given library preparation kit nor any of the other reagents. Our aim was solely to advocate for the use  of a standardized protocol, which includes the use of an automated procedure to minimize the impact of experimenter-derived variability. Along the same lines, paraformaldehyde cross-linking conditions were those previously used for various types of assays, including the ChIP-seq of transcription factors. This being said, we do agree with the reviewer that shorter crosslinking times are currently used by several laboratories; nevertheless we would like to emphasize that 30 minutes paraformaldehyde fixation did not prohibit multiple assays to perform with the top level "triple A" quality.

We have also included modifications in the Conclusion part of the manuscript concerning the evaluation of antibody batches. Indeed, in the same manner that different antibodies sources can affect with the performance of the assay, we believe that different batches of polyclonal antibodies may present variable quality performances. However, it is difficult to perform population studies using public data in the context of antibody batches, as there is no systematic information about the antibody batch in public databases. In this context, we want to point out that the scientific community would benefit significantly if repositories like GEO would ask authors at the time of submission to include a mandatory description, of the experiments corresponding to the datasets provided, including among others the target, cell line or tissue, the antibody source and batch, and all other relevant experimental descriptions.
